# Virulence-related genotypic differences among *Bacillus cereus* ocular and gastrointestinal isolates and the relationship to endophthalmitis pathogenesis

**DOI:** 10.3389/fcimb.2023.1304677

**Published:** 2023-12-01

**Authors:** Phillip S. Coburn, Frederick C. Miller, Austin L. LaGrow, Huzzatul Mursalin, Anna Gregory, Aaron Parrott, Daniel Astley, Michelle C. Callegan

**Affiliations:** ^1^ Department of Ophthalmology, University of Oklahoma Health Sciences Center, Oklahoma City, OK, United States; ^2^ Department of Cell Biology, University of Oklahoma Health Sciences Center, Oklahoma City, OK, United States; ^3^ Department of Family and Preventive Medicine, University of Oklahoma Health Sciences Center, Oklahoma City, OK, United States; ^4^ Department of Microbiology and Immunology, University of Oklahoma Health Sciences Center, Oklahoma City, OK, United States; ^5^ Dean McGee Eye Institute, Oklahoma City, OK, United States

**Keywords:** endophthalmitis, bacillus, infection, ocular, gastrointestinal

## Abstract

**Background:**

*Bacillus cereus (Bc)* can cause self-limiting gastrointestinal infections, but when infecting the eye, can cause rapid and irreversible blindness. This study investigated whether clinical ocular and gastrointestinal *Bc* isolates differed in terms of virulence-related genotypes and endophthalmitis virulence.

**Methods:**

Twenty-eight *Bc* ocular, gastrointestinal, and laboratory reference isolates were evaluated. Hemolysis assays were performed to assess potential differences in hemolytic activity. The presence of twenty *Bc* virulence-related genes was assessed by PCR. A subset of ocular and gastrointestinal isolates differing in PCR positivity for 5 virulence genes was compared to strain ATCC14579 in an experimental murine model of endophthalmitis. At 8 hours post infection, retinal function was evaluated by electroretinography, and intraocular bacterial concentrations were determined by plate counts.

**Results:**

Gastrointestinal *Bc* isolates were more hemolytic than the *Bc* ocular isolates and ATCC14579 (p < 0.0001). *Bc* ocular isolates were more frequently PCR-positive for *capK, cytK, hblA, hblC*, and *plcR* compared to the gastrointestinal isolates (p ≤ 0.0002). In the endophthalmitis model, mean A-wave retention did not differ significantly between eyes infected with ATCC14579 and eyes infected with the selected ocular or gastrointestinal isolates (p ≥ 0.3528). Similar results were observed for mean B-wave retention (p ≥ 0.0640). Only one diarrheal isolate showed significantly greater B-wave retention when compared to ATCC14579 (p = 0.0303). No significant differences in mean A-wave (p ≥ 0.1535) or B-wave (p ≥ 0.0727) retention between the selected ocular and gastrointestinal isolates were observed. Intraocular concentrations of ATCC14579 were significantly higher than the selected ocular isolate and 3 of the gastrointestinal isolates (p ≤ 0.0303). Intraocular concentrations of the selected ocular isolate were not significantly different from the gastrointestinal isolates (p ≥ 0.1923).

**Conclusions:**

Among the subset of virulence-related genes assessed, 5 were significantly enriched among the ocular isolates compared to gastrointestinal isolates. While hemolytic activity was higher among gastrointestinal isolates, retinal function retention and intraocular growth was not significantly different between the selected ocular and gastrointestinal isolates. These results suggest that *Bc* strains causing gastrointestinal infections, while differing from ocular isolates in hemolytic activity and virulence-related gene profile, are similarly virulent in endophthalmitis.

## 1 Introduction

Endophthalmitis is an intraocular infection that poses a significant threat to vision. It can occur after ocular surgery (referred to as postoperative endophthalmitis or POE), following a traumatic penetrating injury to the globe (known as posttraumatic endophthalmitis or PTE), or as a result of microorganisms crossing the blood retinal barrier following hematogenous spread from another infection site in the body (referred to as endogenous endophthalmitis or EE) (Goldberg et al., 1991; Callegan et al., 1999a; Callegan et al., 2007; Lemley and Han, 2007; Coburn and Callegan, 2012; Durand, 2013; Jackson et al., 2014; Astley et al., 2016; Parkunan and Callegan, 2016; Miller et al., 2019). Posttraumatic endophthalmitis (PTE) occurs in 3-17% of cases involving open globe injuries, and constitutes approximately 25%-30% of all cases of exogenous endophthalmitis (Bhagat et al., 2011). *Bacillus cereus* (*Bc*) is among the most devastating etiological agents of posttraumatic endophthalmitis (PTE), characterized by severe inflammation, poor visual prognoses, and, in certain instances, the necessity to remove the affected eye (Miller et al., 2008; Dave et al., 2018). *Bc* endophthalmitis is particularly difficult to treat due to the rapid progression of the infection (Miller et al., 2008; Dave et al., 2018). The significant damage to the eye and poor visual outcomes during *Bc* endophthalmitis likely results from a combination of mechanisms involving bacterial virulence-related factor production and host immune response to these factors (Callegan et al., 1999a; Callegan et al., 2007; Coburn and Callegan, 2012; Durand, 2013; Jackson et al., 2014; Astley et al., 2016; Parkunan and Callegan, 2016; Miller et al., 2019). The importance of *Bc* virulence-related factors was evident in the observation that the absence of a subset of these factors, controlled by the PlcR/PapR transcriptional regulatory system, resulted in a marked reduction in the virulence of endophthalmitis (Callegan et al., 2003).

*Bc* is also recognized as one of the leading causes of bacterial gastrointestinal (GI) illness. The illnesses caused by *Bc* are typically classified as either emetic intoxication, caused by the production of cereulide, a heat-stable toxin in foods left unattended or stored improperly, or as diarrheal infection, caused by enterotoxins produced in the small intestine of the host (Ehling-Schulz et al., 2004; Ehling-Schulz et al., 2006; Bottone, 2010; Granum, 2017; Dietrich et al., 2021; Enosi Tuipulotu et al., 2021). The isolates responsible for *Bc* GI infections produce a variety of toxins that contribute to the pathogenesis of these infections; however, in contrast to ocular infections, *Bacillus* GI infections are often self-limiting after 24 hours requiring no medical attention. If medical attention is required, care is generally supportive (Bottone, 2010). In the current study, we hypothesized that the increased severity of ocular infections when compared to the relatively milder GI diseases may be in part due to differences in the types of virulence factors carried by ocular isolates as compared to GI isolates.

To address this hypothesis, we investigated a set of clinical ocular and GI isolates for the presence of a subset of virulence-related genes to which we previously ascribed roles in endophthalmitis, and/or determined were expressed in explanted vitreous or *in vivo*. We previously evaluated the contribution of the multi-component toxin hemolysin BL (Hbl), the metalloproteases InhA1, InhA2, and InhA3, and the master virulence regulator PlcR to the pathogenesis of endophthalmitis. In a rabbit model, we did not detect differences between a wild type strain of *Bc* and the isogenic *hbl* mutant derivative strain in terms of infection progression and severity (Callegan et al., 1999b). However, Beecher et al. observed significant retinal architecture disruption and neutrophil infiltration after direct injection of purified Hbl (1995). In a mouse model, we demonstrated that a mutant lacking *inhA1*, *inhA2*, and *inhA3* was significantly less virulent than the parental wildtype *Bacillus* strain (Livingston et al., 2021). We also showed that PlcR contributes to disruption of an *in vitro* blood retinal barrier (Moyer et al., 2008), and to the severity of inflammation and loss of retinal function in a mouse model (Callegan et al., 2003). Taken together, these results suggested important roles for these genes in governing the severity of *Bc* endophthalmitis. Therefore, the presence or absence of these genes might distinguish ocular from GI isolates.

To identify additional genes that might contribute to *Bc* virulence in endophthalmitis, and with a view towards identification of potential targets for therapeutic intervention, we assessed expression levels of a set of *Bc* virulence-related genes after growth to stationary phase in explanted rabbit vitreous, as well as at 8 hours post infection in a murine model of endophthalmitis (Coburn et al., 2020; Coburn et al., 2021). Expression of *hbl*, *inhA1*, *inhA2*, *clo* (cereolysin O), *entC* (enterotoxin C)*, nheA* and *nheB* (nonhemolytic enterotoxin Nhe), and *sodA1* and *sodA2* (manganese-based superoxide dismutases SodA1 and SodA2) were all detected in *ex vivo* vitreous at stationary phase, and at 8 hours post infection *in vivo*. Interestingly, expression of the transcriptional regulatory genes *plcR*, *sinR*, and *sinI* was detected *in ex vivo* vitreous, but not *in vivo* at this time point (Coburn et al., 2020; Coburn et al., 2021). Expression of the *slpA* gene (surface layer protein A), that we identified as an important contributor to inflammation and retinal function loss in a mouse model of endophthalmitis, was detected *in vivo* (Coburn et al., 2021). Based on these results, we sought to determine if *Bc* clinical ocular and GI isolates differed in positivity for these endophthalmitis-related virulence genes. Differences in the types of virulence factors employed by different strains might explain the more robust inflammatory response seen in the eye compared to other anatomic sites such as the GI tract. Here, we demonstrated that among a subset of 20 virulence-related genes, only 5 were significantly enriched in ocular isolates relative to GI isolates. However, these virulence-related genotypic profile differences did not translate to demonstrable differences between an ocular isolate and GI isolates in infection severity in a mouse model of endophthalmitis.

## 2 Materials and methods

### 2.1 Bc strains, media, and reagents

Twenty-eight *Bc* isolates were investigated in this study (**Table 1**). ATCC 14579 is a prototypical strain with a sequenced and annotated genome, and we have extensively characterized this strain in our murine endophthalmitis model in terms of infection outcomes and severity (Astley et al., 2016; Coburn et al., 2020; Coburn et al., 2021). In the current study, this strain served as the reference, control strain for *in vitro* and *in vivo* experiments, and hereafter is referred to as *Bc*41. While this strain was originally a dairy isolate, because of its extensive characterization and use in the laboratory, we classified it as a laboratory strain. We also included two additional laboratory strains as part of our analysis (*Bc*82, *Bc*83). The clinical ocular isolates included seven isolated from patients with posttraumatic endophthalmitis (PTE) (*Bc*4, *Bc*8, *Bc*64, *Bc*65, *Bc*66, *Bc*67), three from patients with blepharitis (BLE) (*Bc*54, *Bc*56, *Bc*57), three from patients with infected contact lenses (CL) (*Bc*55, *Bc*58, *Bc*59), one from a keratitis patient (KER) (*Bc*63), and one from a patient diagnosed with endogenous endophthalmitis (EE) (*Bc*144). The GI isolates include five diarrheal isolates (*Bc*122, *Bc*123, *Bc*124, *Bc*125, *Bc*130), three emetic isolates (*Bc*126, *Bc*127, *Bc*128), and an atoxic gastrointestinal isolate (*Bc*129). For the *in vitro* hemolysis assay, total genomic DNA purification, and the murine endophthalmitis model, all isolates were grown in brain heart infusion (BHI) broth at 37°C for 18 h. Bacterial concentrations at 18 hours were determined by serial dilutions of these cultures and plating on BHI agar to ensure similar levels of growth.

**Table 1:** Clinical ocular, GI, and laboratory isolate sources and virulence-related gene profiles.

	Isolates	Type	Reference	*sodA1*	*sodA2*	*inhA1*	*inhA2*	*inhA3*	*hblA*	*hblC*	*hblD*	*cytK*	*clo*	*entC*	*capK*	*fur*	*sinI*	*sinR*	*plcR*	*nheA*	*nheB*	*nheC*	*slpA*
**Ocular**	***Bc*4**	**PTE**	**DMEI**	**•**	•	•	•	•	•	•	•	•	•	•	•	•	•	•	•	•	•	•	•
	***Bc*8**	**PTE**	**DMEI**	**•**	•	•	•	•	•	•	•	•	•	•	•	•	•	•	•	•	•	•	•
	***Bc*54**	**BLE**	**a**	**•**	•	•	•	•	•	•	•	•	•	•	•	•	•	•	•	•	•	•	
	***Bc*55**	**CL**	**a**	**•**	•	•	•	•	•	•	•	•	•	•	•	•	•	•	•	•	•	•	
	***Bc*56**	**BLE**	**a**	**•**	•		•	•	•	•	•	•		•	•		•	•	•	•	•	•	
	***Bc*57**	**BLE**	**a**	**•**	•	•	•	•	•	•	•	•	•	•	•	•	•	•	•	•	•	•	
	***Bc*58**	**CL**	**a**	**•**	•	•	•	•	•	•	•	•	•	•	•	•	•	•	•	•	•	•	
	***Bc*59**	**CL**	**a**	**•**	•	•	•	•	•	•	•	•	•	•	•	•	•	•	•	•	•	•	
	***Bc*63**	**KER**	**COML**	**•**		•	•	•	•	•	•	•	•	•	•	•	•	•	•	•	•	•	
	***Bc*64**	**PTE**	**COML**	**•**	•	•	•	•	•	•	•	•	•	•	•	•	•	•	•	•	•	•	
	***Bc*65**	**PTE**	**COML**	**•**	•	•	•	•	•	•	•	•	•	•	•	•	•	•	•	•	•	•	
	***Bc*66**	**PTE**	**COML**	**•**	•	•	•	•	•	•	•	•	•	•	•	•	•	•	•	•	•	•	
	***Bc*67**	**PTE**	**COML**	**•**	•	•	•	•	•	•	•	•	•	•	•	•	•	•	•	•	•	•	
	***Bc*68**	**KER**	**COML**	**•**	•	•	•		•	•	•	•	•	•	•	•	•	•		•	•	•	
	***Bc*96**	**PTE**	**DMEI**	**•**	•	•	•	•	•	•	•	•	•	•	•	•	•	•	•	•	•	•	
	***Bc*144**	**EE**	**DMEI**	**•**	•	•	•	•	•	•	•	•	•	•	•	•	•	•	•	•	•	•	
**Gastrointestinal**	***Bc*122**	**Diarrheal**	**FDA**	**•**	•	•	•	•			•		•	•		•	•	•		•	•	•	
	***Bc*123**	**Diarrheal**	**FDA**	**•**	•	•	•	•			•		•	•		•	•	•	•	•	•	•	
	***Bc*124**	**Diarrheal**	**FDA**	**•**	•	•	•	•			•		•	•		•	•	•		•	•	•	
	***Bc*125**	**Diarrheal**	**FDA**	**•**	•	•	•	•			•		•	•		•	•	•		•	•	•	
	***Bc*126**	**Emetic**	**FDA**	**•**	•	•	•	•			•		•	•		•	•	•		•	•	•	
	***Bc*127**	**Emetic**	**FDA**	**•**	•	•	•	•			•		•	•		•		•		•	•	•	
	***Bc*128**	**Emetic**	**FDA**	**•**	•	•	•	•			•		•	•		•	•	•		•	•	•	
	***Bc*129**	**Atoxic**	**FDA**	**•**	•	•	•	•			•		•	•		•	•	•		•	•	•	
	***Bc*130**	**Diarrheal**	**FDA**	**•**	•	•	•	•	•	•	•	•	•	•	•	•	•	•	•	•	•	•	
**Lab**	***Bc*41**	**ATCC**	**b**	**•**	•	•	•	•	•	•	•	•	•	•	•	•	•	•	•	•	•	•	•
	***Bc*82**	**LAB**	**c**	**•**	•	•	•	•					•	•		•	•	•		•	•	•	
	***Bc*83**	**LAB**	**c**	**•**	•	•	•	•					•	•		•	•	•		•	•	•	

a Callegan et al., 2006.

b Astley et al., 2016; Coburn et al., 2020; Coburn et al., 2021.

c Senesi et al., 2002.

Isolates that were PCR positive using primers specific for each virulence-related gene are indicated by dots. Highlighted strains were used in the in vivo portion of the study. Abbreviations for isolate types are as follows: BLE, blepharitis; CL, contact lens; EE, endogenous endophthalmitis; KER, keratitis; PTE, posttraumatic endophthalmitis; LAB, laboratory. Reference/source abbreviations are as follows: DMEI, Dean McGee Eye Institute; COML, Campbell Ophthalmic Microbiology Laboratory; FDA, U.S. Food and Drug Administration.

### 2.2 *In vitro* hemolysis assay

All *Bc* isolates were grown as described above and centrifuged at 4,300 x g for 10 min to pellet bacteria. Supernatants were filter sterilized with a 0.22-μm Millex GP filter unit (Merck Millipore Ltd., Tullagreen, Ireland) and immediately placed on ice. Supernatants were then serially diluted 1:2 with phosphate-buffered saline (PBS) pH 7.4. Washed rabbit erythrocytes were added to a final concentration of 5%, and the suspensions incubated for 30 min at 37°C. Unlysed erythrocytes were removed by centrifugation at 500 x g for 5 min. Hemoglobin release was quantified by measuring the optical density at a wavelength of 490 nm using a FLUOstar Omega microplate spectrophotometer (BMG Labtech, Inc., Cary, NC). Values are expressed as percentage of hemolysis relative to a 100% lysis control in which 5% rabbit erythrocytes were lysed in water. Values represent the mean ± standard error of the mean (SEM) from three independent experiments.

### 2.3 Virulence-related gene profiling

Genomic DNA was isolated from 18 h cultures of all *Bc* isolates using standard methods (McBride et al., 2009). Primers specific to virulence-related *Bc* genes (**Table 2**) were designed based on the *Bc*41 (ATCC 14579) genome (Accession # NC_004722.1). PCR amplifications were performed using 100 ng of purified genomic DNA from each isolate as template, and the GoTaq^®^ Flexi DNA Polymerase (Promega, Madison, WI). PCR conditions were optimized for each primer pair. PCR products were analyzed by electrophoresis through standard 1% agarose gels. Genomic DNA from *Bc*41 served as the positive control for all reactions, and reactions without template DNA served as negative controls.

**Table 2:** Sequences of primers used in this study.

Primer	Sequence
capKFor	5’-CGCAAATTCTTGTTGGGAAT-3’
capKRev	5’-TCGACATGTTCACCTCGATA-3’
cloFor	5’-GAGAGGATCCCCTTGCATGTTTATTAGTTAGTC-3’
cloRev	5’-GAGATCTAGAGTTAGGTAGTTCAGCACTTACCG-3’
cytKFor	5’-CTGGCGCTAGTGCAACATTA-3’
cytKRev	5’-TTGCAGTTCCGAATGTGAAG-3’
entCFor	5’-AACTAAAGCTCGCGAAGCAG-3’
entCRev	5’-GCGATTGCTACTCCGTAACC-3’
furFor	5’-TGAACGAATTAAGAAGCAATTACA-3’
furRev	5’-CCCATGGAATGTTAAACGATG-3’
hblAFor	5’-TGCGAGGTGAAATTCAACAA-3’
hblARev	5’-TCAATATGCCCTAGAACGCC-3’
hblCFor	5’ACTCTCGCAACACCAATCGT-3’
hblCRev	5’-TGCTCGCTGTTCTGCTGTTA-3’
hblDFor	5’-CACAAGAAACGACCGCTCAA-3’
hblDRev	5’TTTGCGCCCATTGTATTCCA-3’
inhA1For	5’-ATTCATGCAGGTGTTGGACA-3’
inhA1Rev	5’-AATTGTGGCGTACCTTCTGC-3’
inhA2For	5’-GACCTGGAATCGTTCGTGTT-3’
inhA2Rev	5’-GCAGTGTCAGAACCAGCGTA-3’
inhA3For	5’-GACCTGGAATCGTTCGTGTT-3’
inhA3Rev	5’-GCAGTGTCAGAACCAGCGTA-3’
nheAFor	5’-GGTTACAGCAGTATCTACGAGTTGC-3’
nheARev	5’-GTCGCCTCTGCTTCAGTTTGTGATA-3’
nheBFor	5’-GCTCTATCAGCACTGATGGCAGTAT-3’
nheBRev	5’-GTAGCAATAACTACTGCACCACCGAT-3’
nheCFor	5’-GACCAGCAGGATTCCAAGATGTAAT-3’
nheCRev	5’-GCTGCATCAATTGTTTCTGTCATGT-3’
plcRFor	5’-AGAATTGAATCGGGTGCGGT-3’
plcRRev	5’-TGCATCTTCAACCTCTGCCC-3’
sinIFor	5’-CGCACTGGATCAAGAATGG-3’
sinIRev	5’-GGATGGCTTCATACGTTGG-3’
sinRFor	5’-AAAGCTGGCGTTGCTAAATC-3’
sinRRev	5’-GGAGACACCAGAGTTCATTGC-3’
slpAFor	5’-GTGCGAAGTACGTATTTAGTCCTCA-3’
slpARev	5’-CGATTCGTTCGAATCCAGGAATCTC-3’
sodA1For	5’-GAGAGGATCCCGCCGATGTAGTCTGGACGA-3’
sodA1Rev	5’-GAGAAAGCTTCCCTTATGCGTATGATGCTT-3’
sodA2For	5’-GAGAGAATTCGACTATGATGAACTAGAGCCAC-3’
sodA2Rev	5’-GAGAGGATCCGGTAACAAACTCGGGCGCC-3’

### 2.4 Mice

All procedures described in this study were performed in accordance with the recommendations in the Guide for the Care and Use of Laboratory Animals, the ARVO Statement for the Use of Animals in Ophthalmic and Vision Research, and the policies set forth by the University of Oklahoma Health Sciences Center Institutional Animal Care and Use Committee. *In vivo* experiments were performed with 8 to 10-week old C57BL/6J mice (cat. no. 000664) purchased from The Jackson Laboratory (Bar Harbor, ME, USA). Mice were housed under biosafety level 2 micro isolation conditions on a 12-hour on/12-hour off light cycle. Mice were acclimated for at least 2 weeks prior to intraocular infection to equilibrate their microbiota and to allow for physiological and nutritional stabilization.

### 2.5 Experimental *Bc* endophthalmitis

Mice were anesthetized with a combination of ketamine (Ketamine HCl, 85 mg/kg body weight; Covetous, Portland, ME, USA) and xylazine (AnaSed, 14 mg/kg body weight; Akorn, Decatur, IL, USA). Intravitreal injections were performed with sterile borosilicate glass micropipettes (Kimble Glass Company, Vineland, NJ, USA) beveled to an approximate bore size of 10 to 20 µm (BV-10 KT Brown Type micropipette beveller; Sutter Instrument Company, Novato, CA, USA). Eyes were visualized with a stereomicroscope, and the micropipettes were inserted just posterior to the superior limbus. The right eyes of anesthetized mice were injected with 100 CFU in 0.5 µL, and the left eyes were used as contralateral, uninfected controls (Mursalin et al., 2021b).

### 2.6 Scotopic electroretinography

Prior to scotopic electroretinography (ERG), infected mice were dark adapted for 6 hours. At 8 hours post infection, mice were anesthetized as described above, and topical phenylephrine (10% phenylephrine HCl; Paragon BioTeck, Inc., Portland, OR, USA) to dilate the eyes and topical anesthetic (0.5% proparacaine HCl; Alcon Laboratories, Inc., Fort Worth, TX, USA) were applied to both eyes. Gold-wire electrodes were placed on the cornea of each eye, a reference electrode was attached to the head, and a ground electrode was attached to the tail of the mouse. Eyes were stimulated by the administration of five consecutive white-light flashes (1200 cd·s/m^2^), 60 seconds apart (10-ms duration) to provoke a retinal response. Scotopic A-wave (corresponding to photoreceptor cell activity) and B-wave (corresponding to Müller, bipolar, and amacrine cell activity) amplitudes were recorded for each eye (Espion E2; Diagnosys LLC, Lowell, MA, USA). The percentage of retinal function retained in the infected eye was calculated in comparison with uninfected left eye controls as 100 – {[1 – (experimental A-wave or B-wave amplitude/control A-wave or B-wave amplitude)] × 100} (Mursalin et al., 2021b). Values represent the mean ± standard error of the mean (SEM). Two independent experiments were performed.

### 2.7 Intraocular bacterial quantitation

Following ERG analysis at 8 hours post infection, mice were euthanized by CO_2_ inhalation. The right eyes were enucleated and placed into separate tubes containing 400 µL of sterile PBS and 1.0-mm sterile glass beads (BioSpec Products, Bartlesville, OK, USA). Eyes were then homogenized for 60 seconds at 5000 rpm in a Mini-Beadbeater (BioSpec Products). Eye homogenates were serially diluted 10-fold and plated on BHI agar plates. After overnight incubation, the CFUs per eye were determined as previously described (Mursalin et al., 2021b). Values represent the arithmetic mean ± SEM. Two independent experiments were performed.

### 2.8 Statistics

Data are the arithmetic means ± SEM of all samples in the same experimental group in replicate experiments. Comparative differences between groups were taken to be statistically significant when p < 0.05. Ordinary one-way ANOVA and Brown-Forsythe tests were used to compare differences in hemolytic activity among *Bc* isolates. The Fisher’s Exact test was used to assess differences in virulence-related gene PCR positivity between *Bc* ocular and GI isolates. The Mann–Whitney U test was used to compare experimental groups for ERG and bacterial counts per eye. All statistical analyses were performed using Prism 8.4.3 for Windows (GraphPad, San Diego, CA, USA).

## 3 Results

### 3.1 Hemolytic activity was greater among GI isolates relative to *Bc*41 and clinical ocular isolates

**Figure 1:**
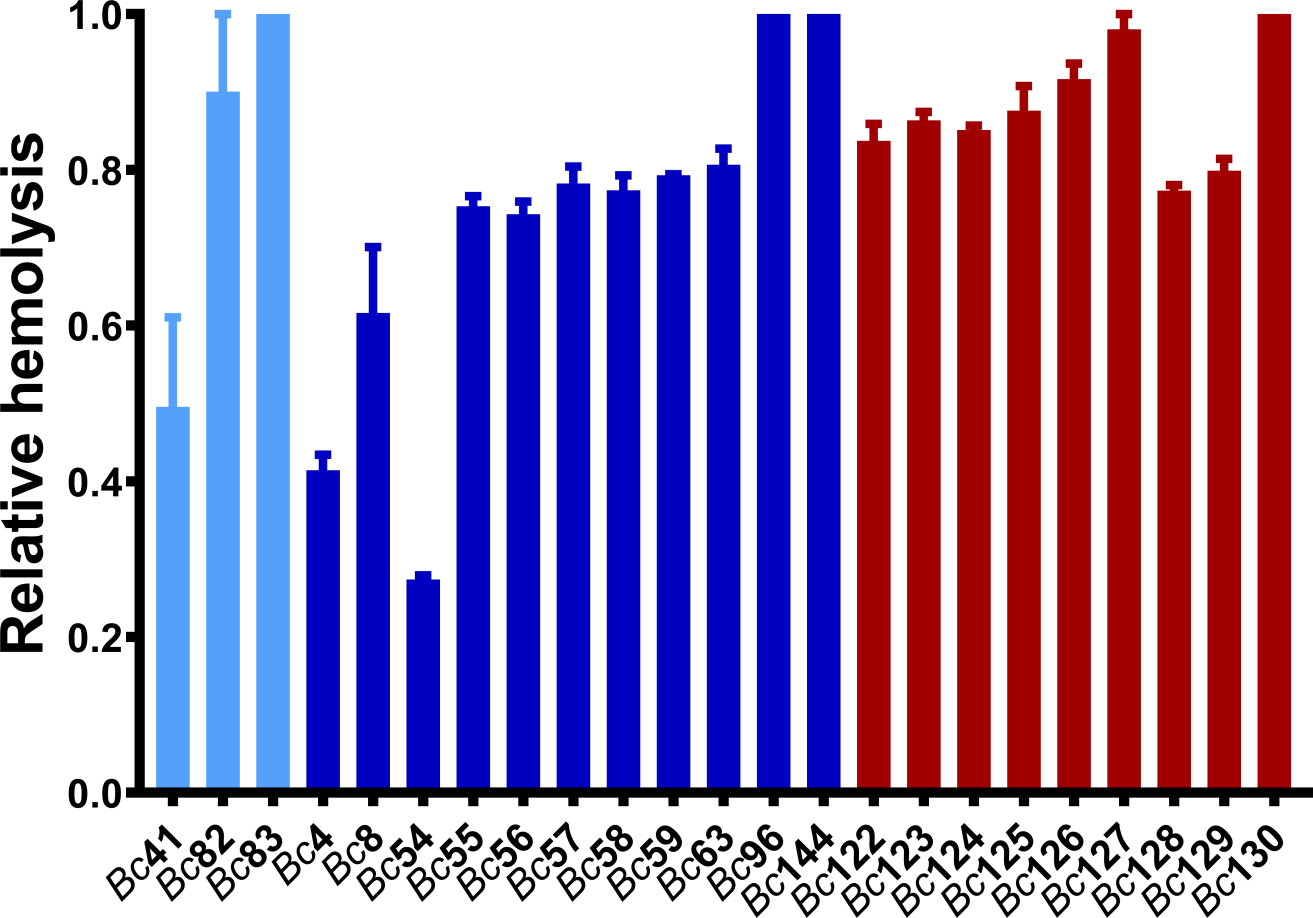
Hemolytic activity of clinical ocular, gastrointestinal, and laboratory isolates. Hemolytic activity of GI isolates was higher than ocular isolates (p < 0.0001). Hemolytic activity of the *Bc* isolates was assessed by incubating filter-sterilized bacterial supernatants with 5% rabbit erythrocytes at 37°C for 30 minutes. Values are expressed as percentage of hemolysis relative to a 100% lysis control in which 5% rabbit RBCs were lysed with sterile water. Values represent the means ± SEM from three independent experiments. Light blue bars are *Bc*41 (ATCC 14579) and laboratory isolates, dark blue bars are ocular isolates, and red bars are GI isolates.

To begin to determine whether GI and ocular isolates differed in terms endophthalmitis pathogenesis and virulence, we first assessed the *in vitro* hemolytic activity of 28 *Bc* isolates (**Table 2**) after cultivation in brain-heart infusion media (BHI) for 18 hours. While variation in hemolytic activity was observed among these isolates (**Figure 1**), GI isolates as a group were significantly more hemolytic against rabbit erythrocytes than ocular isolates (p < 0.0001). This suggested that the GI isolates might actually be more toxic in the eye than ocular isolates.

### 3.2 PCR positivity for the virulence-related genes *capK, cytK, hblA, hblC*, and *plcR* was higher among ocular isolates than GI isolates

In our previous studies, we analyzed the *in vitro* and *in vivo* expression of set of *Bc* virulence-related genes by RNA-Seq to potentially identify targets for novel endophthalmitis therapies (Coburn et al., 2020; Coburn et al., 2021). In the current study, we evaluated GI and ocular isolates for differences in PCR positivity for a subset of 16 of these genes that were expressed in *ex vivo* vitreous or in a murine model of endophthalmitis (Coburn et al., 2020; Coburn et al., 2021). These included *clo* (cereolysin O), *entC* (putative enterotoxin), *hblA, hblC*, and *hblD* (components of tripartite pore-forming hemolysin BL toxin), *nheA* and *nheB* (lytic components of the nonhemolytic enterotoxin), *inhA1, inhA2*, and *inhA3* (secreted zinc metalloproteases), *sodA1* and *sodA2* (manganese-based superoxide dismutases), *slpA* (surface layer protein A), *plcR* (master toxin and virulence gene regulator), and *sinI* and *sinR* (phase regulators). We also assessed four virulence-associated genes that were not included in our previous studies, but that have been implicated in *Bc* virulence. These included *capK* (capsule biosynthesis protein K), *cytK* (cytotoxin K), *fur* (ferric uptake regulator), and *nheC* (binding component of the tripartite nonhemolytic enterotoxin). PCR positivity for each of the 20 genes for all isolates is shown in **Table 1**. The percentage of isolates that were PCR positive for 15 of the 20 genes was similar between ocular and GI isolates for (**Table 3**) (p > 0.05). The percentage of ocular isolates positive for five genes was significantly higher than the percentage of GI isolates positive for those genes (**Table 3**) (p ≤ 0.0002). These included *capK* (100% in ocular versus 11.1% in GI), *cytK* (100% in ocular versus 11.1% in GI), *hblA* (100% in ocular versus 11.1% GI), *hblC* (100% in ocular versus 11.1% in GI), and *plcR* (93.8% in ocular versus and 22.2% in GI). Each of these genes was amplifiable in 93.8% or greater of ocular isolates, and in 22.2% or less of GI isolates. These results suggested that the presence of these genes might contribute to enhanced virulence of ocular isolates in endophthalmitis relative to gastrointestinal isolates.

**Table 3:** Cumulative PCR positivity percentages among *Bc* isolates for the subset of virulence-related genes evaluated in this study.

Gene	Gene ID #	*Bc* ATCC 14579 ID #	Ocular isolates (% PCR Positive)	Gastrointestinal isolates (% PCR Positive)	p-value
*capK*	1203933	BC1584	100	11.1	**<0.0001**
*clo*	1207442	BC5101	93.8	100	>0.05
*cytK*	1203459	BC1110	100	11.1	**<0.0001**
*entC*	1203164	BC0813	100	100	>0.05
*fur*	1206436	BC4091	93.8	100	>0.05
*hblA*	1205449	BC3102	100	11.1	**<0.0001**
*hblC*	1205451	BC3104	100	11.1	**<0.0001**
*hblD*	1205450	BC3103	100	100	>0.05
*inhA1*	1203633	BC1284	93.8	100	>0.05
*inhA2*	1203017	BC0666	100	100	>0.05
*inhA3*	1205332	BC2984	93.8	100	>0.05
*nheA*	1204158	BC1809	100	100	>0.05
*nheB*	1204159	BC1810	100	100	>0.05
*nheC*	1204160	BC1811	100	100	>0.05
*plcR*	1207690	BC5350	93.8	22.2	**0.0002**
*sinI*	1203632	BC1283	100	88.9	>0.05
*sinR*	1203631	BC1282	100	100	>0.05
*slpA*	1202823	BC0470	12.5	0	>0.05
*sodA1*	1206617	BC4272	100	100	>0.05
*sodA2*	1207785	BC5445	93.8	100	>0.05

P-values in bold indicate statistical significance.

### 3.3 Retinal function retention was similar between a clinical ocular (PTE) isolate and GI isolates in a mouse model of endophthalmitis

**Figure 2:**
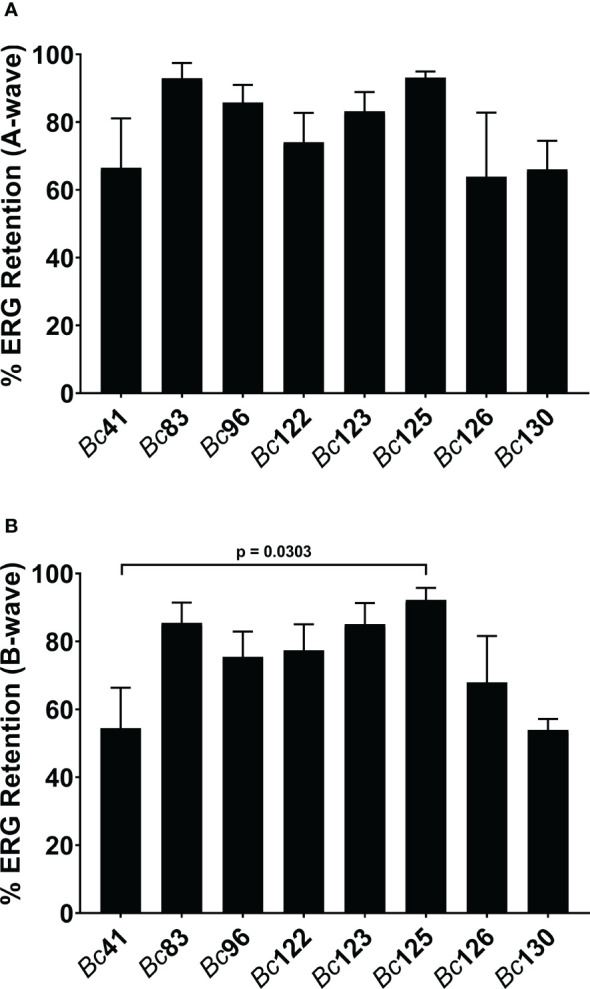
Clinical ocular and gastrointestinal isolates elicited similar retinal function decline. Right eyes of C57BL/6J mice were infected with 100 CFU of either strain *Bc*41 (ATCC 14579), a laboratory isolate (*Bc*83), a PTE isolate (*Bc*96), diarrheal isolates (*Bc*122, *Bc*123, *Bc*125, or *Bc*130), or an emetic isolate (*Bc*126). Retinal function was assessed by electroretinography at 8 hours post infection. Values represent the means ± SEM of *n* ≥ 4 eyes per group in two independent experiments. **(A)** No significant differences were observed in A-wave retention between *Bc*41 and the other isolates, or between *Bc*96 and the GI isolates. **(B)**. No significant differences were observed in B-wave retention between *Bc*41 and the other isolates, with exception of the GI isolate *Bc*125 (p = 0.0303), or between *Bc*96 and the GI isolates.

To determine whether these differences in PCR positivity translated to differences in infection severity in endophthalmitis, a subset of ocular and GI isolates differing in PCR positivity for *capK*, *cytK*, *hblA*, *hblC*, and *plcR* was compared to strain *Bc*41 in a murine model of experimental endophthalmitis. Retinal functional responses of the right, infected eyes relative to the contralateral, control, uninfected eyes were assessed by ERG. At 8 hours post infection, the mean A-wave retention for eyes infected with *Bc*41 was 66.5% (**Figure 2A**). While the mean A-wave retention of eyes infected with the laboratory isolate *Bc*83 was higher at 93.0%, this difference was not statistically significant (p = 0.1134) (**Figure 2A**). The mean A-wave retention of eyes infected with *Bc*41 also did not differ significantly from eyes infected with the PTE isolate *Bc*96 (85.8%; p = 0.4981), or eyes infected with the GI isolates *Bc*122 (74.0%; p = 0.9552), *Bc*123 (83.2%; p = 0.7980), *Bc*125 (93.1%; p = 0.3528), *Bc*126 (63.9%; p = 0.8042), and *Bc*130 (66.1%; p = 0.4343) (**Figure 2A**). The mean A-wave retention of eyes infected with the PTE isolate *Bc*96 did not differ significantly from eyes infected with the GI isolates *Bc*122 (p = 0.2420), *Bc*123 (p = 0.7335), *Bc*125 (p = 0.4514), *Bc*126 (p = 0.5105), or *Bc*130 (p = 0.1535) (**Figure 2A**).

At 8 hours post infection, the mean B-wave retention for eyes infected with *Bc*41 was 54.4% (**Figure 2B**). This did not differ significantly from the mean B-wave retention of eyes infected with *Bc*83 (85.5%; p = 0.0524), *Bc*96 (75.4%; p = 0.2887), *Bc*122 (77.4%; p = 0.2319), *Bc*123 (85.1%; p = 0.0640), *Bc*126 (67.9%; p = 0.7234), or *Bc*130 (53.9%; p = 0.6828) (**Figure 2B**). However, the mean B-wave retention eyes infected with *Bc*41 was significantly lower than eyes infected with the GI isolate *Bc*125 (92.2%; p = 0.0303) (**Figure 2B**). Comparisons of the mean B-wave retention of eyes infected with *Bc*96 with those of eyes infected with the GI isolates revealed no significant differences from *Bc*122 (p = 0.7786), *Bc*123 (p = 0.5664), *Bc*125 (p = 0.3644), *Bc*126 (p = 0.5105), or *Bc*130 (p = 0.0727) (**Figure 2B**). These results demonstrated that intraocular infection with *Bc*41 led to similar A-wave and B-wave amplitude declines as the ocular isolate *BC*96 and the GI isolates *Bc*122, *Bc*123, *Bc*126, and *Bc*130, and only differed in terms of B-wave retention from *Bc*125. Further, infection with *Bc*96 resulted in similar retinal function loss as the GI isolates. These results suggested that PCR profile for these five genes did not influence severity of endophthalmitis at this timepoint, and that the GI isolates, regardless of PCR profile for these genes, were equivalently virulent in a mouse model of endophthalmitis.

### 3.4 Intraocular growth did not differ between a clinical ocular (PTE) isolate and GI isolates in a mouse model of endophthalmitis

**Figure 3:**
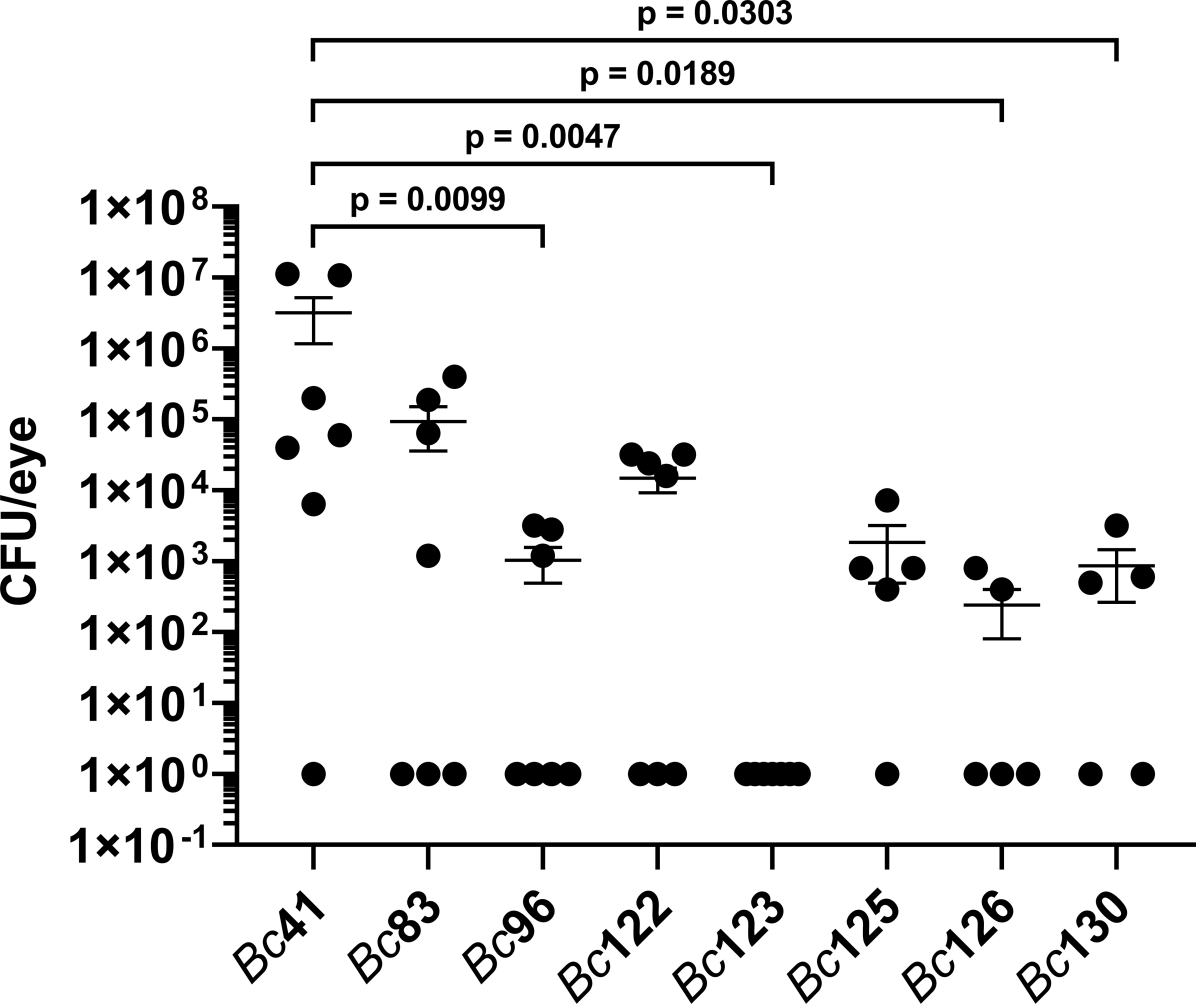
Intraocular growth of the clinical ocular isolates was similar to that of GI isolates. Right eyes of C57BL/6J mice were infected with 100 CFU of either strain *Bc*41 (ATCC 14579), a laboratory isolate (*Bc*83), a PTE isolate (*Bc*96), diarrheal isolates (*Bc*122, *Bc*123, *Bc*125, or *Bc*130), or an emetic isolate (*Bc*126). Eyes were harvested at 8 hours post infection, and intraocular bacteria quantified. Values represent the means ± SEM of *n* ≥ 5 eyes per group in two independent experiments. Intraocular concentrations of *Bc*41 were significantly higher than *Bc*96 (p = 0.0099), *Bc*123 (p = 0.0047), *Bc*126 (p = 0.0189), and *Bc*130 (0.0303). No significant differences were observed between *Bc*96 and any of the GI isolates (p ≥ 0.1923). Note that all data points shown on the graph with a value of 1 CFU/eye represented mouse eyes in which no bacteria were detected after plating the entire volume of eye homogenate and therefore the actual concentration was 0 CFU/eye. These 0 values were set to a value of 1 for plotting on a logarithmic scale.

To assess whether differences in the PCR positivity profiles for *capK*, *cytK*, *hblA*, *hblC*, and *plcR* genes influences intraocular growth, bacterial concentrations were measured after infection with *Bc*41, *Bc*83, *Bc*96, *Bc*122, *Bc*123, *Bc*125, *Bc*126, and *Bc*130. At 8 hours post infection, the mean concentration of *Bc*41 was 3.2 x 10^6^ CFU/eye (**Figure 3**). This was significantly higher than the mean concentration of the PTE isolate *Bc*96 (1.0 x 10^3^ CFU/eye; p = 0.0099), and the mean concentrations of the GI isolates *Bc*123 (0 CFU/eye; p = 0.0047), *Bc*126 (2.4 x 10^2^ CFU/eye; p = 0.0189), and *Bc*130 (8.6 x 10^2^ CFU/eye; p = 0.0303) (**Figure 3**). In the case of *Bc*123, no bacteria were detected in any of the infected mouse eyes after plating the entire volume of the eye homogenate. However, for graphical representation on a logarithmic scale, the zeroes were set to a value of 1. No significant differences were detected between the mean concentration of *Bc*41 and the mean concentration of the laboratory strain *Bc*83 (9.3 x 10^4^ CFU/eye; p = 0.2716), or the mean concentrations of the GI isolates *Bc*122 (1.5 x 10^4^ CFU/eye; p = 0.0542) and *Bc*125 (1.8 x 10^3^ CFU/eye; p = 0.0543). Intraocular growth of the PTE isolate *Bc*96 was not significantly different from any of the GI isolates (p ≥ 0.1923) (**Figure 3**). Interestingly, while the PCR profile for *Bc*96 was identical to *Bc*41 for these five genes, and was also identical for 19 of the 20 genes under consideration with exception of *slpA*, intraocular growth of *Bc*96 was significantly lower than *Bc*41. *Bc*41 also grew to significantly higher concentrations than the GI isolates *Bc*123, *Bc*126, and *Bc*130. While *Bc*41 was PCR positive for all five of these genes, *Bc*123 was positive for only *plcR*, *Bc*126 was negative for all five genes, and *Bc*130 was positive for all 5 genes and thus its profile was identical to *Bc*41. Moreover, while the PCR profiles for these genes was different between *Bc*96 and the GI isolates, there were no significant differences in intraocular growth at 8 hours post infection. These results suggested that the PCR positivity profile for these 5 genes did not influence intraocular growth.

## 4 Discussion

*Bc* is a leading cause of intraocular infection that results in uniformly poor visual prognosis regardless of medical intervention. *Bc* endophthalmitis is particularly difficult to treat due to the rapid nature of the disease, often leading to significant vision loss within 24 hours (Goldberg et al., 1991; Callegan et al., 1999a; Callegan et al., 2007; Lemley and Han, 2007; Miller et al., 2008; Bhagat et al., 2011; Coburn and Callegan, 2012; Durand, 2013; Jackson et al., 2014; Astley et al., 2016; Parkunan and Callegan, 2016; Dave et al., 2018; Miller et al., 2019). Vision loss during endophthalmitis progression is a result of damage to the non-regenerative neural tissue of the eye brought about by a combination of the host immune response and bacterial virulence factor production (Goldberg et al., 1991; Callegan et al., 1999a; Callegan et al., 2007; Lemley and Han, 2007; Miller et al., 2008; Bhagat et al., 2011; Coburn and Callegan, 2012; Durand, 2013; Jackson et al., 2014; Astley et al., 2016; Parkunan and Callegan, 2016; Dave et al., 2018; Miller et al., 2019). The *Bc* genome possesses a plethora of virulence-related factors that have been experimentally demonstrated or hypothesized to contribute to disruption of retinal architecture and function through direct toxicity towards retinal cells, and/or indirectly by eliciting a potent inflammatory response. We have previously demonstrated that the absence of toxins regulated by the PlcR/PapR transcriptional regulatory system significantly blunts the severity of *Bc* endophthalmitis (Callegan et al., 2003). However, the absence of the chemokines CXCL1, CXCL2, CXCL10, CCL2, or CCL3 resulted in significantly improved retinal function and decreased inflammation following *Bc* infection (Parkunan et al., 2016; Mursalin et al., 2021a; Mursalin et al., 2022). These studies pointed to the importance of both *Bc* virulence-related factors and to host innate immune response in mediating the severity of *Bc* endophthalmitis.

*Bc* is also a well-recognized causative agent of bacterial GI intoxications and infections. Emetic intoxications, characterized by nausea and vomiting, result from the consumption of food containing pre-formed cereulide toxin produced by *Bc* following contamination and vegetative growth in improperly prepared or stored food (Ehling-Schulz et al., 2004; Ehling-Schulz et al., 2006; Bottone, 2010; Granum, 2017; Dietrich et al., 2021; Enosi Tuipulotu et al., 2021). Gastrointestinal illness, characterized by diarrhea and abdominal cramps, results from consumption of food contaminated with live *Bc* cells or spores (Ehling-Schulz et al., 2004; Ehling-Schulz et al., 2006; Bottone, 2010; Granum, 2017; Dietrich et al., 2021; Enosi Tuipulotu et al., 2021). Gastrointestinal disease manifestations are linked to the production of the enterotoxins hemolysin BL (Hbl), non-hemolytic enterotoxin (Nhe), and cytotoxin K (CytK) in the small intestine (Dietrich et al., 2021). Both types of GI illnesses are often self-limiting after 24 hours and do not require medical intervention. If medical attention is required, care is generally supportive (Bottone, 2010). However, in stark contrast to these types of infections, the entry of *Bc* into the eye results in a rapid pro-inflammatory response, destruction of retinal tissue, and poor visual prognoses. The possibility arises that in addition to host anatomical site, variation in strains causing these types of infections in terms of virulence-related genotypes might account for the differences in severity and outcome. Therefore, we hypothesized that strains causing GI illnesses differ in terms of virulence-related factor genotypes than strains causing endophthalmitis.

To begin to address this hypothesis, the PCR positivity of a subset of virulence-related genes was determined for a set of clinical ocular isolates, including those obtained from posttraumatic and endogenous endophthlamitis cases, and compared to the PCR positivity of these genes for a set of both emetic and diarrheal gastrointestinal isolates. This subset of genes was selected based on our previous studies that experimentally demonstrated their contribution to endophthalmitis and/or demonstrated expression in an ocular infection-related environment, explanted vitreous, or *in vivo* (Callegan et al., 1999b; Callegan et al., 2003; Moyer et al., 2008; Coburn et al., 2020; Coburn et al., 2021; Livingston et al., 2021). Among the exotoxins produced by *Bc*, we evaluated the genes encoding hemolysin BL based on its links to both gastrointestinal disease and endophthalmitis (Beecher et al., 1995; Dietrich et al., 2021). While we previously did not detect differences between a wildtype strain of *Bc* and the isogenic *hbl* mutant derivative strain in terms of infection progression and severity in a rabbit model of endophthalmitis (Callegan et al., 1999b), Beecher and colleagues observed that direct intravitreal injection of purified Hbl toxin resulted in significant retinal architecture disruption and elicited significant neutrophil infiltration (Beecher et al., 1995). Taken together, these results suggested that in the absence of other *Bc* factors, Hbl was capable of directly damaging the retina and inciting an inflammatory response, and that the lack of observable differences between the wildtype and *hbl* mutant strains might have been due to the presence of other *Bc* toxins. The *Bc* immune inhibitor A genes, *inhA1*, *inhA2*, and *inhA3*, have been hypothesized to contribute to *Bc* virulence several different mechanisms including degradation of host immune mediators, acquisition of nutrients by degrading extracellular matrices, and disruption of tissue barriers (Livingston et al., 2021). In a mouse model of endophthalmitis, we previously demonstrated that a triple mutant lacking *inhA1*, *inhA2*, and *inhA3* was significantly less virulent than the parental wildtype *Bacillus* strain (Livingston et al., 2021). Intraocular growth of the triple mutant was significantly lower than the wildtype strain at 12, 14, and 16 hours post infection, and was undetectable at 16 hours. Retinal function retention was significantly higher than the wildtype strain at 12, 14, and 16 hours (Livingston et al., 2021). These results suggested a significant role for these metalloproteases in intraocular virulence. We also showed that PlcR contributes to disruption of an *in vitro* blood retinal barrier (Moyer et al., 2008), and to the severity of inflammation and loss of retinal function in a mouse model (Callegan et al., 2003). Taken together, these results suggested important roles for these genes in governing the severity of *Bc* endophthalmitis. Therefore, the presence or absence of these genes might distinguish ocular from GI isolates.

In our previous work, we quantified *in vitro* and *in vivo* expression levels of *hblA*, *hblC*, *hblD*, *plcR*, and *inhA1*, *inhA2*, and *inhA3*, as well as additional virulence-related genes (Coburn et al., 2020; Coburn et al., 2021). The results of these analyses served as the basis of our rationale for selecting them as potentially distinguishing clinical ocular from GI isolates (Coburn et al., 2020; Coburn et al., 2021). Expression levels were determined in both an ocular infection-related environment, explanted vitreous, and in an experimental murine endophthalmitis model by RNA-Seq (Coburn et al., 2020; Coburn et al., 2021). While expression of the *hbl* genes was detected in *ex vivo* vitreous at stationary phase, and at 8 hours post infection *in vivo*, expression of PlcR was only detected *in ex vivo* vitreous and not *in vivo* at this time point (Coburn et al., 2020; Coburn et al., 2021). In addition, expression of *inhA1* and *inhA2* was detected after growth in *ex vivo* vitreous to stationary phase, and *in vivo* at 8 hours post infection (Coburn et al., 2020; Coburn et al., 2021). However, *inhA3* expression was not detected in either environment (Coburn et al., 2020; Coburn et al., 2021). We also detected expression of the genes encoding cereolysin O (*clo*), enterotoxin C (*entC*), the nonhemolytic enterotoxin Nhe (*nheA* and *nheB*), and the manganese-based superoxide dismutases SodA1 and SodA2 (*sodA1* and *sodA2*) in both *ex vivo* vitreous and *in vivo* (Coburn et al., 2020; Coburn et al., 2021). Expression of the surface layer protein A (*slpA*) was detected *in vivo* (Coburn et al., 2021). Interestingly, expression of the transcriptional regulatory system SinR/SinI, that contributes to Hbl regulation, was detected in *ex vivo* vitreous at stationary phase, but not at the midpoint of *Bc* infection in our mouse model (Coburn et al., 2020; Coburn et al., 2021). Establishing expression of these genes in relevant ocular environments served as the foundation for additional studies examining the role of these genes in endophthalmitis pathogenesis.

In the current study, we reasoned that differences in the inflammatory response to intraocular versus GI isolates might be at least in part attributable to variation in the armamentarium of *Bc* found in the environment that ultimately cause intraocular or gastrointestinal infections. PCR screening of clinical ocular and GI isolates for the above-mentioned 20 virulence-related genes revealed that ocular isolates more frequently were armed with 5 of these genes than gastrointestinal isolates. Among the genes that were least amplifiable in the GI isolates were *hblA*, *hblC*, *cytK*, *capK*, and *plcR* genes. Interestingly, the laboratory strains shared a similar pattern in PCR amplification with the same genes being the least amplifiable in addition to the gene encoding the third component of the hemolysin BL toxin, *hblD*. Each gene in the ocular isolates was amplifiable in 93.8% or greater of the ocular isolates. Additionally, the *plcR* transcriptional regulator gene was amplifiable in all ocular isolates except 1 isolate from a case of keratitis. In contrast, the *plcR* gene was amplifiable in only 22.2% and 33.3% of the gastrointestinal and laboratory isolates, respectively. The cytotoxin K-encoding gene *cytK* was amplifiable in all ocular isolates, whereas it was only detectable in 11.1% of the GI isolates. Identical results were obtained for the capsule biosynthesis protein K-encoding gene *capK*. For all virulence-related genes evaluated, among the ocular isolates, 11 out of 16 were PCR-positive for 19 out of the 20 genes. Among endophthalmitis isolates specifically, 2 PTE isolates were PCR-positive for all 20 genes, and 6 were PCR-positive for 19 out of the 20 genes.

The genes encoding hemolysin BL, the nonhemolytic enterotoxin, and cytotoxin K have been associated with the course and severity of diarrheal disease (Dietrich et al., 2021). These genes are enriched among diarrheal disease-associated *Bc* isolates, with the *nhe* gene cluster found in 85%–100*%* isolates, and the *hbl* genes and *cytK-2* detected in 40%–70% of isolates (Ehling-Schulz et al., 2006; Dietrich et al., 2021). In the current study, the frequency of PCR positivity for the *hbl* and *cytK* genes among the GI isolates was lower than these reports, whereas the *nhe* genes were detected in all of the GI isolates. Variation in carriage of the enterotoxin genes among diarrheal isolates has led to the identification of 7 groups, A through G, with group F consisting of isolates positive only for the *nhe* genes (Ehling-Schulz et al., 2006). PCR results from our study suggested that 8 out of 9 GI isolates could be classified as group F, and one isolate, *Bc*130, classified as group A (*hbl*+, *cytK*+, *nhe*+). However, given the high degree of sequence polymorphism observed among the enterotoxin genes (Ehling-Schulz et al., 2006), whole genome sequencing will be necessary to verify these results and conclusively establish the presence or absence of these genes. Sequencing of these isolates will also be necessary to validate carriage of the other virulence-related genes in our study.

The *plcR* gene was amplifiable in 15 out of the 16 ocular isolates, whereas it was only amplifiable in two GI isolates, *Bc*123 and *Bc*130. The PlcR/PapR quorum sensing system responds to cell density and transactivates 45 genes, including virulence-related genes encoding degradative enzymes and toxins, at the beginning of stationary phase (Gohar et al., 2008). Included among these genes are the *hbl* and *nhe* gene clusters, and *cytK*. Expression of the *hbl* and *nhe* genes also involves a myriad of other transcriptional regulatory systems including CodY, ResD, Fnr, CcpA and SinR (Esbelin et al., 2008; Van Der Voort et al., 2008; Esbelin et al., 2009; Fagerlund et al., 2014; Böhm et al., 2016). Less is known regarding the involvement of these other transcriptional control mechanisms in *cytK* expression; however, expression does not seem to involve either CodY or CcpA (Van Der Voort et al., 2008; Böhm et al., 2016). Although *plcR* was not detected in 7 out of the 9 GI isolates that were all positive for the *nhe* genes, it cannot be ruled out that *nhe* expression in these strains, governed by other regulatory systems, might have contributed to the absence of differences in retinal function retention observed between the ocular isolate and GI isolates.

The *capK* gene, encoding a polypeptide putatively involved in capsule biosynthesis, was also significantly enriched in ocular isolates relative to GI isolates. PCR products were obtained for all ocular isolates, whereas only 11.1% of GI isolates were PCR positive for *capK*. The chromosomally-encoded CapK polypeptide from *Bc*41 exhibits similarity to the Wzz protein from *Escherichia coli* (Franco et al., 1998). Wzz in *E. coli* governs O-antigen chain length heterogeneity and has been hypothesized to modulate the O-antigen polymerase, Wzy, leading to variations in the number of O-antigen subunits incorporated into LPS (Bastin et al., 1993). Capsule biosynthesis-related genes, identified in a strain of *Bc* from a pneumonia patient, were demonstrated to contribute to lethality in a mouse model (Scarff et al., 2018). These genes are located on pBC210, one of two plasmids carried by *Bc* strain G9241, an anthrax-like pneumonia isolate, and putatively specify the biosynthesis enzymes for a tetrasaccharide capsule (Scarff et al., 2018). Whether *capK* and capsule production impacts ocular virulence remains to be determined.

Among the genes surveyed in this study, *slpA* was least amplifiable in both ocular and GI tract isolates, with only 2 out 16 of the ocular isolates and none of the GI isolates exhibiting PCR positivity. This is of particular interest in that we have demonstrated that *slpA* functions as an important determinant of *Bacillus* virulence in a mouse model of endophthalmitis. In a *Bacillus thuringiensis* background, deletion of *slpA* resulted in significantly improved retinal function retention and decreased inflammation (Mursalin et al., 2019). Moreover, SlpA was subsequently shown to be a potent activator of both TLR2 and TLR4, and to induce the expression of immune mediators IL-6, TNFα, CCL2, and CXCL-1 *in vitro* (Mursalin et al., 2019; Mursalin et al., 2020a). While the role of *slpA* was ascertained in *B. thuringiensis*, the course and severity of *B. thuringiensis* endophthalmitis in a mouse model is highly similar to that elicited by *Bc*41. Therefore, *slpA* is predicted to contribute to intraocular virulence of *Bc* in an analogous manner, although expression level differences could account for differences in inflammation and severity. The infrequent PCR positivity among our ocular isolates was therefore surprising, and suggests that while *slpA* is an important member of the *Bc* armamentarium, intraocular virulence involves a complex, multifactorial interplay of both *Bc* and host factors.

Although we demonstrated that ocular isolates were significantly more frequently PCR positive for these 5 genes, we did not find significant differences between the ocular isolate *Bc*96 and the GI isolates *Bc*122, *Bc*123, *Bc*126, and *Bc*130 in terms of retinal function retention and intraocular growth. Therefore, observed differences in PCR profile did not translate to demonstrable differences between the isolates of differing origin in intraocular infection outcome. Our results did not support our hypothesis that ocular isolates harbor a subset of certain virulence-related genes that result in more severe intraocular infections than the relatively milder and self-limiting GI infections. Rather, the results suggested that *Bc* isolates, regardless of origin, are still armed with sufficient factors to cause significant damage in the eye. Members of the group *Bc sensu lato* have been divided into three clades (Baldwin, 2020). Clade 1 is comprised of *Bc sensu stricto*, *B. thuringiensis*, and *Bacillus anthracis*. Clade 2 also consists of members of *Bc sensu stricto* and *B. thuringiensis*, and Clade 3 is composed of a diverse array of the other known *Bacillus* species (Baldwin, 2020). Pathogenic members are found in both Clades 1 and 2, with considerable strain to strain variation in pathogenicity and virulence observed (Baldwin, 2020). Aoyagi et al. analyzed 53 *Bc* isolates associated with nosocomial bloodstream infections for the presence of genes related to GI infections, including the *hbl* and *nhe* genes, and *cytK* (2020). Fifty-one out of 53 isolates possessed *nheA*, *nheB*, and *nheC* (Aoyagi et al., 2020), in concordance with other studies that demonstrated a high degree of prevalence of this gene cluster regardless of source of isolation (Enosi Tuipulotu et al., 2021). This finding also agreed with our study, in which all 29 isolates were PCR positive for all three *nhe* genes. In contrast, Aoyagi et al., observed that only 8 out of the 53 isolates were positive for the *hbl* genes, and none of the isolates were positive for *cytK* (2020). Forty-nine of the 53 isolates were classified as Clade 1, with 3 isolates positive for the *hbl* genes, and the other 4 isolates, all *hbl* positive, were categorized as Clade 2 (Aoyagi et al., 2020). The authors also showed that all 4 members of Clade 2 produced enterotoxin by passive latex agglutination assays (Aoyagi et al., 2020). This study agreed with other studies that found a low abundance of the *hbl* genes among Clade 1 members that are associated with bloodstream and nosocomial infections (Horii et al., 2011; Zhang et al., 2011). These results led the authors to postulate that virulence factor profiles of *Bc* strains in Clade 1, that are associated with more severe nosocomial blood stream infections, differ from *Bc* strains in Clade 2, that are associated with GI infections (Aoyagi et al., 2020). In another study, Santos et al. detected *plcR/papR*, *nheA*, *cytK-2*, *plcA*, and *gyrB* more frequently among isolates associated with GI infections and food poisoning than environmental soil and food isolates (Santos et al., 2011). Geographic location and association with food products of animal or plant origin also influenced virulence-related profiles (Berthold-Pluta et al., 2019; Drewnowska et al., 2020). In contrast to our results, these data supported the contention that virulence factor profiles might influence the types of infections caused by *Bc*. It is clear that regardless of genotype, virulence-associated phenotypes can be highly variable, even among strains with identical virulence-related gene profiles (Jessberger et al., 2015; Jessberger et al., 2017). We also observed significant variation in hemolytic activity among our isolates, with GI tract isolates significantly more hemolytic than ocular isolates. However, the degree of hemolytic activity did not correlate with virulence in our endophthalmitis model. This is not surprising given the myriad of *Bc* exotoxins that are also hemolysins and could account for the activity observed in our assay.

We have previously reported that during endophthalmitis progression, *Bc* stimulates activation of both TLR2 and TLR4 pathways, resulting in signal cascade initiation through both MyD88-dependent and MyD88-independent (TRIF-dependent) pathways, respectively (Mursalin et al., 2020a). This results in significant influx of inflammatory cells that contribute to retinal photoreceptor bystander damage and vision loss (Mursalin et al., 2019; Mursalin et al., 2020a). *Bc* is highly immunogenic in this environment, and our results demonstrated that isolates of differing virulence-related gene profile and origin behave equivalently in the eye in terms of endophthalmitis pathogenesis. This suggests that introduction of potentially any *Bc* isolate into the vitreous would result in potential cataclysmic outcomes. These outcomes differ markedly from the other leading Gram-positive causative agents of endophthalmitis that tend to elicit a slower and less robust inflammatory response (Mursalin et al., 2020b). While the host immune status and the unique ocular environment likely contribute to the severity of ocular infections, other yet unidentified ocular virulence-related factors might also contribute to the unique virulence of *Bc* in endophthalmitis. Of particular interest would be to determine whether *Bc* isolates with differing virulence gene-related profiles are more readily treated with antibiotics and anti-inflammatory agents. Future studies will continue to identify virulence factors that contribute to *Bc* endophthalmitis, with the goal of improving extant therapies or identifying novel treatment regimens for this blinding infection.

## Data availability statement

The original contributions presented in the study are included in the article/Supplementary Material. Further inquiries can be directed to the corresponding author.

## Ethics statement

The animal study was approved by The University of Oklahoma Health Sciences Center Institutional Animal Care and Use Committee (IACUC). The study was conducted in accordance with the local legislation and institutional requirements.

## Author contributions

PC: Conceptualization, Data curation, Formal Analysis, Investigation, Methodology, Project administration, Supervision, Validation, Writing – original draft, Writing – review & editing. FM: Conceptualization, Data curation, Formal Analysis, Investigation, Methodology, Validation, Writing – original draft, Writing – review & editing. AL: Data curation, Formal Analysis, Investigation, Methodology, Writing – review & editing. HM: Investigation, Methodology, Validation, Writing – review & editing. AG: Investigation, Methodology, Writing – review & editing. AP: Investigation, Methodology, Writing – review & editing. DA: Investigation, Methodology, Writing – review & editing. MC: Conceptualization, Funding acquisition, Project administration, Resources, Supervision, Writing – review & editing.
